# Ultrasound Assessment of Retained Products of Conception (RPOC): Insights from the Current Literature

**DOI:** 10.3390/jcm14165864

**Published:** 2025-08-19

**Authors:** Giosuè Giordano Incognito, Carla Ettore, Orazio De Tommasi, Roberto Tozzi, Giuseppe Ettore

**Affiliations:** 1 Obstetrics and Gynecology Unit, Maternal Child Department, ARNAS Garibaldi Nesima, 95122 Catania, Italy; cettore@arnasgaribaldi.it (C.E.); giuseppe.ettore@gmail.com (G.E.); 2 Unit of Gynecology and Obstetrics, Department of Women and Children’s Health, University of Padua, 35122 Padua, Italy; orazio.detommasi@studenti.unipd.it (O.D.T.); roberto.tozzi@unipd.it (R.T.)

**Keywords:** retained products of conception, RPOC, ultrasound, endometrial thickness

## Abstract

Retained products of conception (RPOC) represent a significant cause of morbidity in the post-abortive and postpartum periods, potentially leading to abnormal uterine bleeding, pelvic pain, infections, and intrauterine adhesions. Accurate diagnosis is crucial to avoid unnecessary surgical interventions and to preserve future fertility. Transvaginal ultrasound constitutes the primary imaging modality for identifying RPOC, but the lack of standardized diagnostic criteria complicates clinical decision-making. This narrative review explores the current literature on sonographic findings associated with RPOC, focusing on the diagnostic value of endometrial thickness (ET), the presence of intrauterine echogenic masses, and the use of Color Doppler imaging. Although an ET ≥15 mm is frequently used to suspect RPOC, the variability in cut-off thresholds and limited specificity reduce its diagnostic reliability. The detection of an echogenic intrauterine mass appears to be the most sensitive and specific sonographic feature. Color Doppler assessment, particularly the presence of enhanced myometrial vascularity (EMV) and classification systems like the Gutenberg score, offers further insight by stratifying hemorrhagic risk and guiding therapeutic choices. However, vascular parameters such as peak systolic velocity (PSV) and resistive index (RI) demonstrate a substantial overlap between benign and pathological conditions, limiting their stand-alone utility. The review also addresses the differential diagnosis of RPOC, including blood clots, arteriovenous malformations, placental polyps, gestational trophoblastic disease, and endometrial osseous metaplasia. The role of three-dimensional ultrasound remains limited in clinical practice, offering no significant advantage over two-dimensional imaging. Finally, the timing of follow-up ultrasound after medical treatment with misoprostol is critical: delayed assessment reduces overtreatment by allowing time for spontaneous resolution. In conclusion, despite advances in ultrasound technology, the diagnosis of RPOC remains challenging due to heterogeneity in imaging findings and inter-observer variability. A multimodal approach integrating grayscale and Doppler ultrasound with clinical evaluation is essential for optimal management.

## 1. Introduction

Retained products of conception (RPOC) are defined as the persistence of trophoblastic or placental tissue within the uterine cavity following a miscarriage or delivery. The exact prevalence is not clearly defined, but it is estimated that this condition may affect up to 1% of full-term pregnancies and reach 15% in cases of medical abortion [1]. RPOC can present with a wide range of clinical symptoms, including persistent or abnormal uterine bleeding, pelvic pain, fever, and uterine infections. In the long term, inadequate management can promote the formation of intrauterine adhesions, negatively impacting fertility [2]. The treatment of RPOC depends on multiple factors, including the patient’s clinical picture and the ultrasound (US) characteristics. Therapeutic options include conservative treatment, which may be expectant or pharmacological, and invasive procedures such as dilation and curettage (D&C), hysteroscopy, or uterine artery embolization [3]. In more complex cases, particularly in the presence of uncontrollable bleeding, hysterectomy may be necessary [4]. However, although surgical approaches are effective in removing RPOC, they involve non-negligible risks, including uterine perforation, cervical lacerations, infections, and the formation of intrauterine synechiae [5]. Therefore, timely and accurate diagnosis of RPOC is essential both to reduce the risk of such complications and to avoid unnecessary surgical interventions.

Diagnosis is based on clinical evaluation, laboratory tests, and, above all, imaging [6]. Transvaginal US represents the first-line diagnostic method for women with suspected RPOC, proving to be more accurate than clinical assessment alone [7]. The examination relies on the evaluation of several parameters, including endometrial thickness (ET), the presence of an intrauterine echogenic mass, and blood flow assessed via Color Doppler. However, currently there are no universally accepted standardized US criteria for the diagnosis of RPOC, and this lack of consensus has a direct impact on their clinical management. It has been hypothesized that the absence of definitive diagnostic criteria may be attributed to the evolution of US technologies in terms of grayscale and Color Doppler imaging [8]. Furthermore, the US diagnosis of RPOC is subject to significant inter-observer variability. Maslovitz et al. [9] analyzed the histopathological samples of D&C in 69 women with suspected RPOC and demonstrated that the operator’s skill plays a crucial role in the interpretation of US findings. An additional element of complexity is related to the postpartum US appearance of the uterus, which shows considerable individual and physiological variations, making it difficult to distinguish between a normal and a pathological condition [10].

Given the clinical relevance of accurate diagnosis and the ongoing inconsistency in sonographic criteria, a comprehensive synthesis of the literature is necessary to clarify the current evidence. This narrative review aims to critically evaluate the main US parameters used in the diagnosis of RPOC, discussing their diagnostic performance, limitations, and differential considerations, to offer clinicians a clear interpretative framework to support decision-making in daily practice.

## 2. Materials and Methods

**Figure 1:**
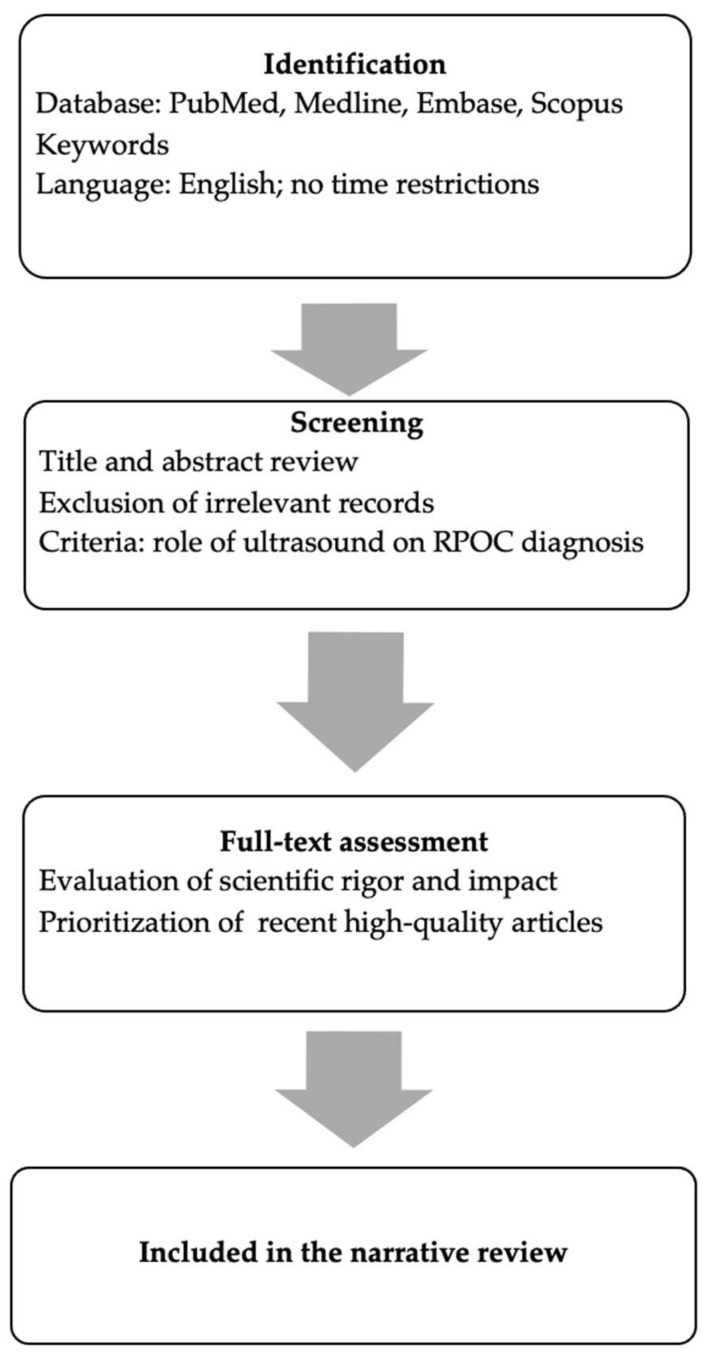
Flow diagram outlining the article selection process. Relevant literature was identified through structured database searches, screened for thematic relevance, and included based on scientific rigor and contribution to the review focus. Abbreviations: RPOC, retained products of conception.

This narrative review was conducted to evaluate the role of the US in the diagnosis of RPOC and to provide a synthesis of the available evidence. The research was conducted using the PubMed, Medline, Embase, and Scopus databases, focusing on studies published from inception to June 2025. The search employed specific keywords to ensure relevance to the area of interest, such as “retained products of conception”, “RPOC”, “residual trophoblastic tissue”, and “placental remnants”, in combination with “ultrasound”, “ultrasonography”, “sonography”, “ecography”, and “TVUS”. Two authors (G.G.I. and C.E.) independently screened titles and abstracts for relevance to the topic. Disagreements during the selection process were resolved through discussion or, when necessary, consultation with a third author (G.E.). All types of peer-reviewed articles written in English were considered, including preclinical studies, clinical studies, reviews, and meta-analyses. Articles were excluded if they were unrelated to the topic of interest or lacked relevance to the role of the US in the diagnosis of RPOC. The selection process prioritized recent publications. References from key review articles were also screened to identify additional relevant sources. A summary of the literature selection process is presented in Figure 1.

## 3. Endometrial Thickness

ET represents one of the main US parameters evaluated in cases of suspected RPOC. However, the cut-off values reported in the literature vary widely, ranging from 10 mm to over 25 mm [11]. An ET of 15 mm or greater is the most frequently used cut-off to suspect the presence of RPOC, as highlighted in the systematic review conducted by Hamel et al. [6]. In this study, more than half of the included papers adopted this threshold to establish diagnostic suspicion in cases of first-trimester pregnancy termination or spontaneous miscarriage occurring before 14 weeks of gestation. However, the diagnostic accuracy of this value remains uncertain: a meta-analysis assessing its reliability did not yield conclusive results, as it was based on only three studies and reported exclusively false-negative cases, i.e., patients who still required surgical revision of the uterine cavity despite having an ET below 15 mm. According to Durfee et al. [12], an ET of less than 10 mm, in the absence of an intrauterine mass, makes the presence of RPOC unlikely. Nevertheless, a reduced thickness does not entirely exclude the diagnosis, since even small RPOC fragments may be clinically relevant. For this reason, Ustunyurt et al. [13] advise against making clinical decisions based solely on ET value, emphasizing the need to consider other US parameters and the patient’s overall clinical picture. Further confirmation of variability in diagnostic criteria emerges from a study that assessed US in the management of first-trimester pregnancy terminations, without defining a specific cut-off for the presence of RPOC [14]. This highlights that, although ET is a frequently used parameter, there is still no unanimous consensus on the most reliable cut-off for diagnosing RPOC.

## 4. Intrauterine Echogenic Mass

The identification of an echogenic mass within the endometrial cavity represents an additional US criterion in the evaluation of RPOC. According to a recent meta-analysis conducted by Sundararajan et al. [11], the presence of an echogenic mass is the US criterion with the highest sensitivity, specificity, and diagnostic predictive value for recognizing RPOC. This mass may conform to the shape of the endometrial cavity or appear as a distinct structure. A key element for correct identification is the presence of a well-defined margin separating it from the endo-myometrial junction. Additionally, it may be surrounded by intracavitary fluid with particulate material, indicative of residual blood. Echogenicity is often heterogeneous, with regular or irregular margins, and may exhibit a lobulated morphology or calcifications.

A prospective study conducted by Matijevic et al. [15] on 93 women also demonstrated that the presence of an endometrial mass is the most sensitive US criterion for the diagnosis of RPOC. The authors defined RPOC as a hyperechoic, hypoechoic, or mixed-pattern mass located within the uterine cavity and with a diameter >10 mm, including both endometrial layers in the mid-sagittal plane. When the ET was less than 10 mm and no mass was present, the diagnosis of RPOC was infrequent. Another study by Mulic-Lutvica and Axelsson [16] defined the echogenic mass as a well-circumscribed formation, often lobulated, possibly with calcifications and lacking fluid components. However, the authors also emphasized that an echogenic mass may be present in asymptomatic postpartum women without actual RPOC.

Shen et al. [17] performed transabdominal US on 39 women with suspected RPOC, based on placental histologic examination showing incomplete fragments. Of these, 21 had an endometrial mass with a thickness >10 mm, and in 15 cases the diagnosis of RPOC was histologically confirmed. Based on these data, the sensitivity of the US for RPOC diagnosis was 93.8%, with a specificity of 73.9%. Neill et al. [18] compared clinical assessment with US in the management of secondary postpartum hemorrhage, noting that the presence of hyperechoic foci had similar sensitivity (93%) but lower specificity (62%). However, they pointed out that the positive predictive value of US for RPOC diagnosis is relatively low (46%). In comparison, the negative predictive value is very high (96%), suggesting that a negative US is highly reassuring.

Finally, a retrospective study [19] of 212 women who underwent hysteroscopy for suspected RPOC demonstrated that an echogenic mass >7 mm had a positive predictive value of 75%, confirming a strong correlation between the US finding and the histological diagnosis. However, the negative predictive value for remnants ≤7 mm was 50%, suggesting that even in the presence of a small echogenic remnant, the possibility of RPOC remains.

These data confirm that the identification of an intrauterine echogenic mass is a relevant diagnostic criterion for RPOC. However, its interpretation must always be integrated with other US parameters and the overall clinical picture of the patient, taking into account the variability of criteria used in the literature to define a suspicious mass and the discrepancies among studies. Furthermore, the absence of such a US finding cannot definitively exclude the diagnosis of RPOC.

## 5. Color Doppler

The use of Color Doppler can provide an additional contribution to the diagnosis of RPOC, as it allows for the assessment of the vascularization of the mass identified through grayscale US. The presence of a vascular signal within the mass increases the positive predictive value, thereby improving diagnostic accuracy. However, according to the meta-analysis by Sundararajan et al. [11], Color Doppler appears to be the least reliable parameter for diagnosing RPOC compared to other US criteria.

A key element in Doppler evaluation is enhanced myometrial vascularity (EMV), which appears as a focal area of increased vascularization extending throughout the full thickness of the myometrium up to the endometrium. The mechanism through which RPOC leads to increased myometrial blood flow is not yet fully understood. It is hypothesized that this may result from the presence of residual vessels diverting arterial flow directly into veins (arteriovenous shunting) or from the secretion of vasoactive substances that inhibit normal myometrial contraction and remodeling of the placental zone [20]. Clinically, this vascular alteration may resolve spontaneously or after removal of the residual tissue [21]. However, this condition is not exclusive to RPOC, as hypervascularization can also be found in other pathologies and represents a common transient phenomenon in the postpartum period [22].

### 5.1. Gutenberg Classification

Color Doppler US was first used by Kamaya et al. [23] to classify RPOC based on their vascularization. The authors divided US findings into four categories, ranging from absence of vascularization (Type 0) to marked hypervascularization (Type 3). This classification was later expanded and adapted, giving rise to the Gutenberg classification [24], which incorporates both Color Doppler vascular assessment and grayscale US characteristics. The Gutenberg classification divides RPOC into four main categories:

- Type 0: hyperechoic avascular mass, with a low probability of persistence;
- Type 1: mass with mixed echogenicity and minimal vascularization, suggestive of organized remnants;
- Type 2: highly vascularized mass, confined to the uterine cavity, with a high risk of bleeding;
- Type 3: highly vascularized mass with myometrial involvement, indicative of a possible risk of arteriovenous malformations (AVMs) or active trophoblastic tissue.

The adoption of this classification plays a crucial role in stratifying bleeding risk and selecting the most appropriate therapeutic strategy. A study conducted by Alonso Pacheco et al. [25] compared hysteroscopic management of patients with Type 0–1 RPOC to those with Type 2–3. The results showed that in patients with Type 2 or 3 RPOC, the use of monopolar energy was necessary to control bleeding during the procedure. In contrast, none of the patients with Type 0 or 1 RPOC required such intervention.

Beyond guiding therapeutic choices, this classification is beneficial in pre-treatment counseling. In cases of Type 0 or 1 RPOC, conservative management with monitoring may be an appropriate option in patients with mild or minimal symptoms. However, when the US shows a Type 2 or 3 RPOC, it is essential to inform the patient about the increased risk of heavy bleeding. Additionally, RPOC in these categories have a higher failure rate with conservative treatments and frequently require hysteroscopic resection for complete resolution.

### 5.2. Internal Myometrial Peak Systolic Velocity (PSV) and Resistive Index (RI)

PSV and RI are hemodynamic parameters assessed by Doppler US to analyze blood flow within vessels. PSV indicates the maximum velocity of blood flow in a vessel during cardiac systole, while RI, calculated using the formula (PSV—End Diastolic Velocity, EDV)/PSV, reflects the degree of vascular resistance in the analyzed area. A high RI value suggests increased resistance to flow and reduced perfusion, whereas a low RI indicates a more continuous and less resistive flow, typical of highly vascularized structures.

In the context of RPOC, Color and Pulsed Doppler are used to assess the vascularization of residual tissue, helping to distinguish benign conditions from pathological ones. Several studies have attempted to identify flow velocity cut-off values in EMV to guide therapeutic decisions, such as choosing between conservative management, surgery, or arterial embolization in suspected RPOC cases.

Timmerman et al. [26], in a series of 30 cases, observed that EMV with a PSV < 39 cm/s was rarely associated with heavy bleeding, whereas PSV values ≥ 83 cm/s could indicate a significant risk of hemorrhage. However, the authors emphasized the need for caution in interpreting these proposed cut-offs, as they were derived from a limited number of patients. Timor-Tritsch et al. [20] recommended stratifying uterine vascular lesions based on PSV, regardless of RPOC presence, suggesting uterine artery embolization in cases with elevated PSV (≥60–70 cm/s).

In a study conducted by Lee et al. [27] on patients with uterine AVMs, including those with concomitant RPOC, PSV values > 76.2 cm/s were reported as potentially indicative of a dangerous AVM, whereas PSV values < 35.8 cm/s appeared to be associated with fewer complications. The authors highlighted that PSV represents a key parameter in multivariate analysis and concluded that Doppler US may be a valuable tool in selecting the most appropriate treatment.

However, a study by Vyas et al. [28] revealed significant overlap in PSV and RI values between patients managed conservatively and those requiring surgical or angiographic intervention. This suggests that these parameters alone are not sufficient to determine the need for invasive treatment definitively. These findings are consistent with other evidence showing no clear correlation between PSV and RI values and the clinical approach adopted [22].

Moreover, an additional diagnostic challenge arises from the fact that the high-velocity, low-resistance flow pattern typical of EMV may be challenging to distinguish from other uterine vascular anomalies using Color Doppler alone. To avoid diagnostic errors and inappropriate treatments, Vyas et al. [28] proposed classifying these postpartum vascular alterations as EMV, regardless of their association with RPOC. This distinction would help prevent confusion with much rarer conditions, such as congenital or acquired AVMs following invasive procedures.

Based on available evidence, the use of internal myometrial PSV and RI in identifying RPOC appears limited, given the significant overlap in values found among patients managed conservatively versus those undergoing active treatment. Although higher PSV cut-off values may suggest an increased risk of bleeding, the interpretation of these parameters should always be contextualized with the clinical picture and other US features. Therefore, the use of PSV and RI alone is not recommended as an exclusive diagnostic criterion for RPOC but rather as a complementary tool alongside US and clinical evaluation.

## 6. Differential Diagnosis

The differential diagnosis of RPOC is essential to distinguish among various intrauterine conditions that may present with similar clinical and US findings. Accurate US assessment, integrated with Color Doppler, is crucial to correctly identify each pathology and determine the most appropriate therapeutic approach.

### 6.1. Intrauterine Blood Clots

Blood clots appear on US as echogenic masses within the endometrial cavity. A distinguishing feature compared to RPOC can be the presence of a clear margin separating the clot from the endo-myometrial junction along its entire circumference. On Color Doppler, clots are typically avascular, showing no blood flow. However, the US appearance may be indistinguishable from that of avascular RPOC. Applying pressure with the US probe can assist in the differential diagnosis: a mobile mass suggests a clot, while an adherent mass points toward a possible RPOC [11].

### 6.2. Uterine Arteriovenous Malformations

Uterine AVMs are characterized by abnormal communications between arteries and veins without the interposition of the regular capillary bed. On the US, they may appear as echogenic masses within the endometrium, similar to RPOC. However, a distinguishing feature is the presence of hypoechoic areas in the myometrium associated with high-velocity, multidirectional flow on Color Doppler. In RPOC, vascularization is generally limited to the endometrium, whereas in AVMs, the abnormal vascular flow primarily involves the myometrium [7]. Recognizing this difference is crucial, as procedures such as D&C, which are indicated for RPOC, may exacerbate bleeding in the presence of an AVM.

### 6.3. Placental Polyp

A placental polyp may be considered a late outcome of an RPOC that, over time, has undergone a process of organization with fibrosis and reduced trophoblastic activity. However, from a clinical and US perspective, the two conditions present different features. RPOC represent a more recent and disorganized residual material that may contain active tissue and cause heavier bleeding. The placental polyp appears on US as a well-circumscribed mass, often with a polypoid or pedunculated appearance, characterized by defined margins and a more compact structure compared to RPOC. On Color Doppler, it may show a uniform and concentrated vascular pattern. Unlike RPOC, which typically manifest earlier with irregular margins, heterogeneous content, and variable vascularization depending on the presence of active trophoblast, the placental polyp tends to be more stable over time, with less pronounced vascularization. This distinction is fundamental for accurate differential diagnosis, as RPOC may resolve spontaneously or require medical or surgical treatment, whereas an asymptomatic placental polyp can be monitored, though persistent bleeding may require hysteroscopy or targeted treatment [29].

### 6.4. Gestational Trophoblastic Disease (GTD)

GTD includes a spectrum of trophoblastic proliferative conditions, ranging from benign forms such as hydatidiform mole to malignant and invasive forms like choriocarcinoma and placental site trophoblastic tumor. Since these conditions can arise after a miscarriage or in the postpartum period, they are part of the differential diagnosis with RPOC. US, combined with serial hCG measurements, is a key tool to distinguish different forms of GTD from RPOC.

In a complete hydatidiform mole, the US shows a hyperechoic intrauterine mass with a disorganized appearance, characterized by numerous small anechoic vesicles that give the typical “snowstorm” pattern, reflecting the absence of normal placental organization. In this form, no embryonic structure or gestational sac is observed, as the embryo does not develop. Amniotic fluid is absent, and the endometrium may appear thickened and heterogeneous. On Color Doppler, there is a hypervascular pattern often described as a “crown” or “ring”, with vessels showing low resistance flow (RI < 0.5) and high-velocity peaks, indicating abnormal trophoblastic proliferation [30].

In a partial hydatidiform mole, US may show a gestational sac containing a fetus, often with severe congenital anomalies and signs of growth restriction. The placenta appears thickened and disorganized, with multicystic areas suggestive of hydropic degeneration of the chorionic villi. Unlike the complete mole, where abnormal trophoblast entirely replaces placental tissue, in the partial mole, it is possible to identify areas of normal placental tissue adjacent to pathological ones. Color Doppler also shows increased trophoblastic vascularization, but with a less homogeneous distribution than in the complete mole [30].

In invasive mole, the abnormal trophoblastic proliferation extends beyond the endometrial cavity, infiltrating the myometrium and potentially involving uterine blood vessels. US reveals a heterogeneous mass with irregular margins, often associated with necrotic areas and cysts within the uterine wall. Color Doppler shows even more pronounced vascularization compared to previous forms, with a diffuse hypervascular pattern and turbulent flow, indicative of trophoblastic invasion of the myometrial vessels [31].

Choriocarcinoma, the most aggressive form of GTD, has a less characteristic US appearance. It generally presents as a hyperechoic intrauterine mass with an irregular and disorganized appearance, lacking the typical cystic pattern seen in molar forms. It may deeply infiltrate the myometrium and give rise to early metastases. On Color Doppler, it shows very high vascularization, with aberrant vessels and arteriovenous shunts resulting in high-velocity, low-resistance flow [32].

The placental site trophoblastic tumor, unlike other forms of GTD, does not exhibit the typical multicystic pattern but instead appears as a solid, hyperechoic intrauterine mass with well-defined margins and more peripheral than central vascularization [33].

### 6.5. Endometrial Osseous Metaplasia

Endometrial osseous metaplasia is a rare condition characterized by the presence of bone tissue within the uterine cavity, often associated with prior abortions. On the US, it appears as hyperechoic structures with posterior acoustic shadowing, suggesting the presence of calcified or osseous material [34]. This presentation is distinctive and differs from RPOC, which generally do not contain numerous bony elements or evident calcifications.

To support the differential diagnosis of vascularized intrauterine findings in the postpartum or post-abortion setting, Table 1 summarizes the main uterine vascular conditions potentially associated with or mimicking retained products of conception, outlining their sonographic and clinical features.

## 7. Three-Dimensional Ultrasound (3D-US)

3D-US has been proposed as a potential tool to improve the diagnosis of RPOC by offering a more detailed volumetric assessment compared to traditional two-dimensional ultrasound (2D-US). However, its actual diagnostic value remains a subject of debate. A study conducted by Belachew et al. [35] evaluated the effectiveness of 3D-US in diagnosing RPOC compared to the traditional 2D technique.

The study included 25 women with postpartum hemorrhage who initially underwent 2D-US, which identified in all but one case the presence of a well-circumscribed echogenic mass, often with a lobulated appearance and calcifications, but lacking fluid components. Subsequently, the same patients were also examined with 3D-US using the Virtual Organ Computer-aided AnaLysis (VOCAL) software to measure the volume of the uterine cavity and compare it with previously collected reference values. Finally, all patients underwent surgical resection of the mass, followed by histological examination to confirm or exclude the presence of RPOC.

The study results showed that an increased uterine cavity volume, assessed with 3D-US, may be indicative of RPOC. However, the authors concluded that 3D-US is not significantly superior to the 2D technique, as it does not provide additional useful information to differentiate RPOC from other intrauterine conditions. Consequently, its use in clinical practice for this indication appears limited.

## 8. Suggested Diagnostic and Management Framework

In the evaluation of suspected RPOC, a structured clinical reasoning that integrates clinical presentation and US findings may assist clinicians in orienting diagnostic interpretation and management decisions. The initial assessment should begin with a detailed clinical evaluation, focusing on the timing relative to delivery or uterine evacuation, presence of abnormal uterine bleeding, pelvic discomfort, or signs of anemia or infection. Transvaginal US represents the first-line instrumental tool, with particular attention to endometrial morphology and vascular features. On grayscale imaging, the detection of intrauterine echogenic or mixed echogenic content, especially when focal and well-defined, may suggest the presence of RPOC. However, this appearance is not specific and may overlap with clots, necrotic tissue, or other uterine abnormalities. In this setting, Doppler interrogation provides complementary information, particularly regarding the degree and pattern of vascularization. The absence of detectable Doppler flow typically supports a conservative approach, especially in hemodynamically stable patients. In such cases, expectant management or medical therapy (e.g., misoprostol) may be considered. In contrast, low-resistance flow or richly vascularized areas may increase suspicion for persistent trophoblastic tissue. When vascularity is localized and moderate, surgical removal may be a suitable option if clinically indicated. However, caution is warranted in cases with marked or atypical vascular patterns (e.g., chaotic flow, serpiginous vessels), which may suggest AVM or other vascular anomalies. In such situations, further imaging with contrast-enhanced ultrasound, magnetic resonance imaging, or computed tomography angiography may be advisable prior to any surgical intervention. Uterine artery embolization remains a key option when abnormal vascular structures are confirmed or in cases of refractory bleeding. Adjunctive laboratory testing, such as serum β-hCG, can provide useful information in select cases, particularly when gestational trophoblastic disease is in the differential diagnosis. Ultimately, management should be tailored to the individual case, balancing clinical stability, imaging findings, reproductive desires, and institutional expertise. In complex or ambiguous scenarios, a multidisciplinary approach involving radiology, gynecology, and interventional services may be essential to ensure optimal outcomes.

## 9. Timing of US Follow-Up After Misoprostol Treatment

A few studies have investigated the most appropriate timing for US reassessment of RPOC following misoprostol treatment for incomplete abortion, and the literature shows wide variability on this matter [6]. A key concept appears to be that the longer the interval between initial treatment and follow-up, the greater the likelihood of spontaneous resolution.

This was demonstrated by the study of Mizrachi et al. [36], which compared early versus delayed follow-up after misoprostol treatment for early pregnancy loss. The results showed that prolonged observation reduces the need for additional interventions, as it allows for the identification of a greater number of complete abortions without intrauterine remnants.

In this context, the MisoREST trial [37] provided further evidence on the importance of timing in the management of RPOC. The study demonstrated that a US performed 6 weeks after the start of treatment confirmed complete expulsion of residual material in 85% of women who, at the 2-week evaluation, still presented an ET greater than 10 mm, considered suggestive of RPOC.

Therefore, performing US follow-up too early may lead to overdiagnosis and unnecessary treatments, while delayed follow-up can support a more conservative approach, reducing the need for invasive interventions.

## 10. Future Directions

One of the major limitations in the US-based diagnosis of RPOC lies in its strong operator dependence. The acquisition and interpretation of US findings are subject to considerable variability depending on the clinician’s experience, training, and technical proficiency. This variability can negatively affect diagnostic accuracy, lead to inconsistent management decisions, and potentially result in both overtreatment and undertreatment. The current literature highlights a lack of universal consensus regarding US criteria, cut-off values, and reporting frameworks. Although some classification systems, such as the Gutenberg score [24], have been proposed to facilitate clinical decision-making, these tools are not yet widely adopted in routine practice. Moreover, no universally accepted guidelines currently provide a standardized approach to the US-based diagnosis of RPOC, and existing protocols often differ significantly between institutions. To address these limitations, future efforts should prioritize the development of structured diagnostic pathways and consensus-based protocols that integrate grayscale and Doppler US findings with clinical evaluation. Standardized training programs and inter-observer reliability studies are also needed to improve reproducibility and minimize subjectivity in image interpretation. In this context, artificial intelligence (AI) represents a promising avenue to enhance diagnostic accuracy and consistency. AI-based tools can assist in image recognition and pattern analysis, supporting clinicians in identifying key US features. Machine learning algorithms trained on large datasets could offer real-time diagnostic support, reduce reliance on operator expertise, and ensure more objective and standardized assessments [38]. While AI integration in gynecologic US is still at an early stage, its future application may contribute significantly to improving the diagnostic reliability of RPOC.

## 11. Conclusions

The US diagnosis of RPOC remains a significant clinical challenge, playing a crucial role in guiding appropriate management and preventing both unnecessary invasive procedures and delayed treatment-related complications. Transvaginal US is the first-line imaging modality due to its accessibility, non-invasiveness, and diagnostic accuracy. However, the lack of universally accepted sonographic criteria limits the standardization of care and contributes to variability in clinical practice. The most frequently evaluated parameters include endometrial thickness, the presence of an intrauterine echogenic mass, and vascularization assessed by Color Doppler. Among these, the detection of an endometrial mass appears to be the most sensitive and specific criterion, whereas endometrial thickness alone has limited diagnostic value. Color Doppler imaging provides additional insights into vascular activity and bleeding risk, supporting clinical decision-making, especially when combined with grayscale ultrasound findings. Despite technological advancements, the interpretation of sonographic findings remains highly operator-dependent, and diagnostic overlap with other intrauterine pathologies often complicates the clinical picture. Therefore, a comprehensive, multimodal approach integrating clinical evaluation, grayscale imaging, and Doppler features is essential to improve diagnostic accuracy and tailor management strategies. Future research should aim to establish standardized sonographic definitions and validate diagnostic algorithms to optimize the management of RPOC and improve patient outcomes.

## Figures and Tables

**Table 1:** Differential diagnosis of vascularized intrauterine conditions in the postpartum or post-abortion uterus.

Condition	Definition	Typical Doppler Features	Anatomical Location	Clinical Implications	Management
RPOC	Residual gestational tissue within the uterine cavity after delivery or miscarriage	Focal intrauterine mass with variable vascularity; low-resistance flow when present	Endometrial cavity	Postpartum bleeding, risk of infection, or incomplete uterine involution	Expectant, medical, uterine artery embolization, or surgical
AVM	Abnormal direct communication between arteries and veins, congenital or acquired	Chaotic, high-velocity turbulent flow; very low resistance; aliasing on color Doppler	Myometrium	Risk of severe bleeding; may be life-threatening	Uterine artery embolization
EMV	Increased myometrial vascularity, often secondary to postpartum changes or RPOC	Prominent vessels at endo-myometrial junction; low-resistance, high-velocity flow	Endo-myometrial interface	Often transient; can persist after RPOC resolution	Expectant; resolves spontaneously or after RPOC removal
Subinvolution of the placental site	Delayed regression of placental bed vessels	Dilated, tortuous vessels with slow flow	Inner myometrium near placental site	Prolonged bleeding, often misdiagnosed	Expectant or embolization in select cases
Uterine pseudoaneurysm	Focal outpouching of an artery due to trauma (e.g., surgery, curettage)	To-and-fro flow pattern; single vascular cavity with yin-yang sign	Myometrial artery	Risk of rupture and acute hemorrhage	Embolization or surgical
Highly vascularized placental polyp	Benign fibrous residual tissue with persistent vascular stalk	Single feeding vessel	Endometrial cavity	Chronic spotting or bleeding	Expectant management or hysteroscopic removal
Choriocarcinoma	Highly malignant trophoblastic tumor arising after any type of pregnancy	Marked hypervascularity with chaotic flow; low-resistance, high-velocity signals	Endometrium or invading myometrium	Rapidly progressive disease; distant metastases common	Chemotherapy ± surgery
Placental site trophoblastic tumor	Rare neoplasm from intermediate trophoblasts at implantation site	Moderate peripheral vascularity	Endo-myometrial or myometrial	Can present with abnormal bleeding; potential for local invasion	Surgery ± chemotherapy

Abbreviations: AVM, arteriovenous malformations; EMV, enhanced myometrial vascularity; RPOC, retained products of conception.

## Data Availability

Not applicable.
